# Crystal structure of 3-methyl­pyridinium picrate: a triclinic polymorph

**DOI:** 10.1107/S2056989015017090

**Published:** 2015-09-17

**Authors:** Jeganathan Gomathi, Doraisamyraja Kalaivani

**Affiliations:** a PG and Research Department of Chemistry, Seethalakshmi Ramaswami College, Tiruchirappalli 620 002, Tamil Nadu, India

**Keywords:** crystal structure, 3-methyl­pyridinium picrate, triclinic polymorph, π–π stacking, anti­convulsant activity.

## Abstract

The title mol­ecular salt, C_6_H_8_N^+^·C_6_H_2_N_3_O_7_^−^ (systematic name: 3-methyl­pyridinium 2,4,6-tri­nitro­phenolate), crystallizes in the triclinic space group *P*-1. The crystal structure of the monoclinic polymorph (space group *P*2_1_/*n*) has been reported [Stilinovic & Kaitner (2011 ▸). *Cryst. Growth Des.* **11**, 4110–4119]. In the crystal, the anion and cation are linked *via* bifurcated N—H⋯(O,O) hydrogen bonds, enclosing an *R*_1_^2^(6) graph-set motif. These units are linked *via* C—H⋯O hydrogen bonds, forming a three-dimensional framework. Within the framework there are π–π inter­actions present, involving inversion-related picrate anions and inversion-related pyridinium cations, with inter-centroid distances of 3.7389 (14) and 3.560 (2) Å, respectively.

## Abstract 2

In the crystal of the title mol­ecular salt, the 3-methyl­pyridinium cation and the picrate anion are linked *via* bifurcated N—H⋯(O,O) hydrogen bonds, forming an 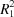
(6) ring motif. These units are linked *via* C—H⋯O hydrogen bonds, forming a three-dimensional framework·The compound exhibits anti­convulsant activity.

## Chemical context   

Stilinovic & Kaitner (2011 ▸) have synthesized a series of 20 crystalline picrates of pyridine derivatives and through single crystal X-ray diffraction studies revealed the presence of a common synthon. They reported the crystal structure of the monoclinic polymorph of the title mol­ecular salt: space group *P*2_1_/*n*.
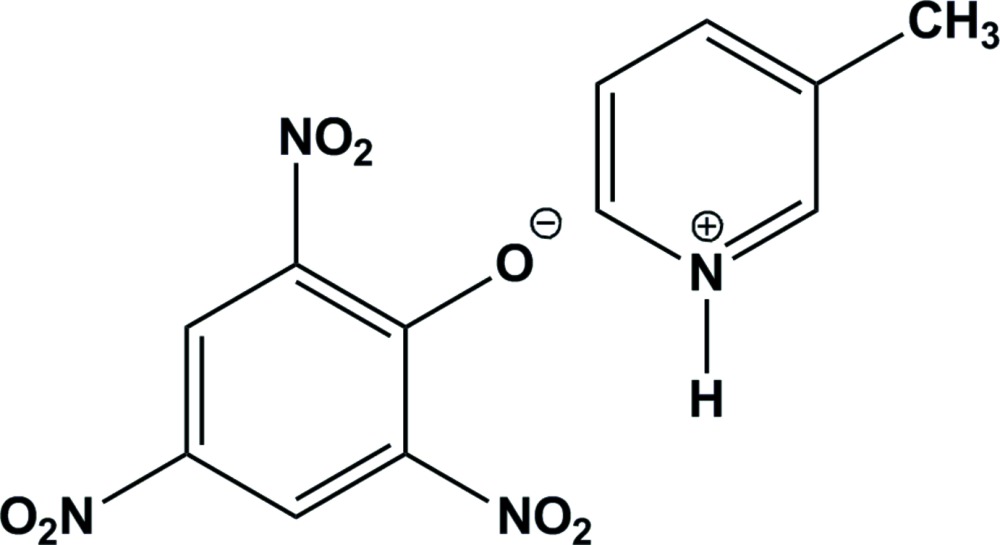


The observation that the presence of more than one heterocyclic component in a mol­ecule enhances the biological response and thermal stability encouraged us to synthesize several new carbon-bonded anionic sigma complexes from chloro­nitro-aromatic compounds and pyrimidine derivatives in the presence of pyridine bases (Babykala *et al.*, 2014 ▸; Buvaneswari & Kalaivani, 2013 ▸; Mangaiyarkarasi & Kalaivani, 2013 ▸; Manickkam & Kalaivani, 2011 ▸, 2014 ▸; Sridevi & Kalaivani, 2012 ▸). Surprisingly, when we made an attempt to synthesize a similar type of complex from the electron-deficient chloro­nitro­aromatic compound (picryl chloride), an imidazole derivative (hydantoin) and 3-methyl­pyridine, the title salt crystallized from the medium (ethanol) instead of the expected carbon-bonded anionic sigma complex.

## Structural commentary   

**Figure 1:**
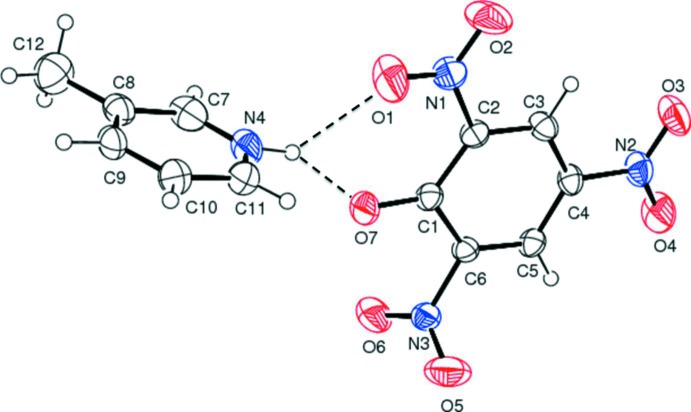
A view of the mol­ecular structure of the title mol­ecular salt, with atom labelling. Displacement ellipsoids are drawn at the 40% probability level. Hydrogen bonds are shown as dashed lines (see Table 1 ▸).

The mol­ecular structure of the title mol­ecular salt is shown in Fig. 1 ▸. The anion and cation are linked *via* bifurcated N—H⋯(O,O) hydrogen bonds, enclosing an 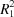
(6) graph-set motif (Fig. 1 ▸ and Table 1 ▸). In the picrate anion, the two nitro groups flanking the C—O^−^ bond are oriented differently. Nitro group O1/N1/O2, involved in N—H⋯O hydrogen bonds as noted above, is inclined to the benzene ring by 6.7 (3)°, while nitro group O5/N3/O6 is inclined to the benzene ring by 70.07 (3)°, probably to alleviate steric crowding. The third nitro group (O3/N2/O4), *para* with respect to the C—O^−^ bond, deviates only slightly from the benzene ring, making a dihedral angle of 6.6 (3)°.

## Supra­molecular features   

**Figure 2:**
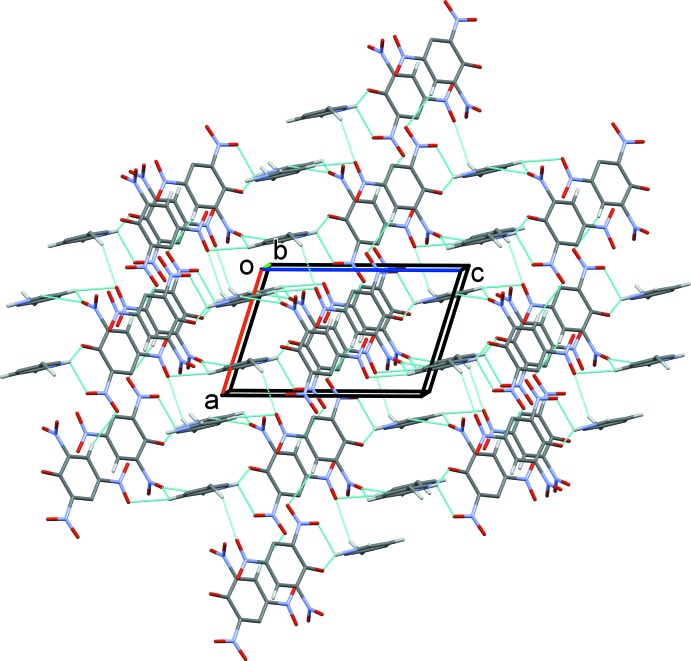
A view along the *b* axis of the crystal packing of the title mol­ecular salt. Hydrogen bonds are shown as dashed lines (see Table 1 ▸), and H atoms not involved in these inter­actions have been omitted for clarity.

In the crystal, the anionic and cationic hydrogen-bonded units are linked *via* C—H⋯O hydrogen bonds, forming a three-dimensional structure (Figs. 2 ▸ and 3 ▸, and Table 1 ▸). Within this framework there are slipped parallel π–π inter­actions present, involving inversion-related picrate anions [inter-centroid distance = 3.7389 (14) Å, inter-planar distance = 3.5829 (8) Å, slippage = 1.069 Å] and inversion-related pyridinium cations [inter-centroid distance = 3.560 (2) Å, inter-planar distance = 3.5548 (14) Å, slippage = 0.422 Å].

## Anti­convulsant activity   

The anti­convulsant activity of synthesized 3-methyl­pyridinium picrate has been measured by employing the maximal electro shock (MES) method (Bhattacharya & Chakrabarti, 1998 ▸; Misra *et al.*, 1973 ▸). Different stages of convulsion such as tonic flexion, tonic extensor, clonus convulsion, stupor and recovery/death have been examined. Though all phases are reduced, noticeable decrease is observed in the clonus phase and hence the title mol­ecule may be a potent agent for controlling myoclonic type epilepsy in the future.

## Database survey   

A search of the Cambridge Structural Database (Version 5.36, last update May 2015; Groom & Allen, 2014 ▸) yielded 40 hits for *meta*- or *para*-substituted pyridinium picrate salts. In the picrate anions, the average dihedral angle of the nitro group *para* to the C—O^−^ bond with respect to the benzene ring is *ca* 6°, while for the two nitro groups on either side of the C—O^−^ bond the dihedral angles are both *ca* 26–28°. In the title compound, the latter two dihedral angles are quite different being 6.7 (3) and 70.07 (3)°. In the monoclinic polymorph (UBEFEO; Stilinovic & Kaitner, 2011 ▸), these three dihedal angle are *ca* 3.60, 6.92 and 13.83°, respectively, and the cation and anion are also linked *via* bifurcated N—H⋯(O,O) hydrogen bonds.

## Synthesis and crystallization   

Picryl chloride [2.56 g (0.01 mol)] was dissolved in 30 ml of rectified spirit and mixed with hydantoin [1.00 g (0.01 mol)] in 30 ml of rectified spirit. After mixing these solutions, 3 ml of 3-methyl­pyridine (0.03 mol) was added and the temperature of the mixture was raised to 313 K. At this temperature, the mixture was stirred for 5 to 6 h. The solution was then cooled to room temperature, filtered and the filtrate kept at 298 K. After a period of 4 weeks, dark shiny maroon-red–coloured crystals formed from the solution. The crystals were filtered, powdered and dried. The dry solid was washed with 50 ml of dry ether (5 ml for each aliquot) and recrystallized from rectified spirit (yield: 60%; m.p. 483 K).

## Refinement   

Crystal data, data collection and structure refinement details are summarized in Table 2 ▸. The NH H atom was located in a difference Fourier map and freely refined. The C-bound H atoms were included in calculated positions and refined as riding: C—H = 0.93–0.96 Å with *U*_iso_(H) = 1.2*U*_eq_(C).

## Supplementary Material

Crystal structure: contains datablock(s) global, I. DOI: 10.1107/S2056989015017090/su5205sup1.cif

Structure factors: contains datablock(s) I. DOI: 10.1107/S2056989015017090/su5205Isup2.hkl

Supporting information file. DOI: 10.1107/S2056989015017090/su5205Isup3.cml

CCDC reference: 1417794

Additional supporting information:  crystallographic information; 3D view; checkCIF report

## Figures and Tables

**Figure 3:**
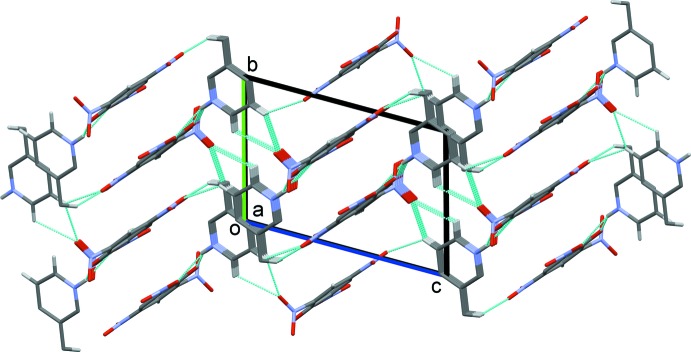
A view along the *a* axis of the crystal packing of the title mol­ecular salt. Hydrogen bonds are shown as dashed lines (see Table 1 ▸), and H atoms not involved in these inter­actions have been omitted for clarity.

**Table 1:** Hydrogen-bond geometry (, )

*D*H*A*	*D*H	H*A*	*D* *A*	*D*H*A*
N4H4*A*O1	0.93(4)	2.27(4)	2.827(4)	118(4)
N4H4*A*O7	0.93(4)	1.79(5)	2.638(4)	152(4)
C5H5O2^i^	0.93	2.51	3.406(4)	162
C10H10O3^ii^	0.93	2.55	3.220(4)	129
C12H12*B*O3^iii^	0.96	2.56	3.414(5)	149

Symmetry codes: (i) 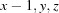; (ii) 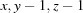; (iii) 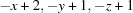.

**Table 2:** Experimental details

Crystal data	
Chemical formula	C_6_H_8_N^+^C_6_H_2_N_3_O_7_
*M*_r_	322.24
Crystal system, space group	Triclinic, *P* 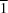
Temperature (K)	293
*a*, *b*, *c* ()	8.1224(5), 8.7016(5), 11.3161(6)
, , ()	98.239(3), 100.318(3), 117.635(3)
*V* (^3^)	673.17(7)
*Z*	2
Radiation type	Mo *K*
(mm^1^)	0.13
Crystal size (mm)	0.35 0.30 0.25

Data collection	
Diffractometer	Bruker Kappa APEXII CCD
Absorption correction	Multi-scan (*SADABS*; Bruker, 2004 ▸)
*T*_min_, *T*_max_	0.952, 0.969
No. of measured, independent and observed [*I* > 2(*I*)] reflections	13299, 2374, 1717
*R*_int_	0.029
(sin /)_max_ (^1^)	0.595

Refinement	
*R*[*F* ^2^ > 2(*F* ^2^)], *wR*(*F* ^2^), *S*	0.045, 0.131, 1.07
No. of reflections	2374
No. of parameters	212
H-atom treatment	H atoms treated by a mixture of independent and constrained refinement
_max_, _min_ (e ^3^)	0.24, 0.25

Computer programs: *APEX2*, *SAINT* and *XPREP* (Bruker, 2004 ▸), *SIR92* (Altomare *et al.*, 1993 ▸), *ORTEP-3 for Windows* (Farrugia, 2012 ▸), *Mercury* (Macrae *et al.*, 2008 ▸), *SHELXL2014* (Sheldrick, 2015 ▸) and *PLATON* (Spek, 2009 ▸).

## supplementary crystallographic information

### Crystal data

**Table:**

C_6_H_8_N^+^·C_6_H_2_N_3_O_7_^−^	*Z* = 2
*M**_r_* = 322.24	*F*(000) = 332
Triclinic, *P*1	*D*_x_ = 1.590 Mg m^−^^3^
*a* = 8.1224 (5) Å	Mo *K*α radiation, λ = 0.71073 Å
*b* = 8.7016 (5) Å	Cell parameters from 5179 reflections
*c* = 11.3161 (6) Å	θ = 2.7–26.9°
α = 98.239 (3)°	µ = 0.13 mm^−^^1^
β = 100.318 (3)°	*T* = 293 K
γ = 117.635 (3)°	Block, red
*V* = 673.17 (7) Å^3^	0.35 × 0.30 × 0.25 mm

### Data collection

**Table:**

Bruker Kappa APEXII CCD diffractometer	2374 independent reflections
Radiation source: fine-focus sealed tube	1717 reflections with *I* > 2σ(*I*)
Graphite monochromator	*R*_int_ = 0.029
ω and φ scan	θ_max_ = 25.0°, θ_min_ = 2.7°
Absorption correction: multi-scan (*SADABS*; Bruker, 2004)	*h* = −9→9
*T*_min_ = 0.952, *T*_max_ = 0.969	*k* = −10→10
13299 measured reflections	*l* = −13→13

### Refinement

**Table:**

Refinement on *F*^2^	0 restraints
Least-squares matrix: full	Hydrogen site location: mixed
*R*[*F*^2^ > 2σ(*F*^2^)] = 0.045	H atoms treated by a mixture of independent and constrained refinement
*wR*(*F*^2^) = 0.131	*w* = 1/[σ^2^(*F*_o_^2^) + (0.0452*P*)^2^ + 0.5934*P*] where *P* = (*F*_o_^2^ + 2*F*_c_^2^)/3
*S* = 1.07	(Δ/σ)_max_ < 0.001
2374 reflections	Δρ_max_ = 0.24 e Å^−^^3^
212 parameters	Δρ_min_ = −0.25 e Å^−^^3^

### Special details

**Table:**

Geometry. All esds (except the esd in the dihedral angle between two l.s. planes) are estimated using the full covariance matrix. The cell esds are taken into account individually in the estimation of esds in distances, angles and torsion angles; correlations between esds in cell parameters are only used when they are defined by crystal symmetry. An approximate (isotropic) treatment of cell esds is used for estimating esds involving l.s. planes.

### Fractional atomic coordinates and isotropic or equivalent isotropic displacement parameters (Å^2^)

**Table:**

	*x*	*y*	*z*	*U*_iso_*/*U*_eq_	
C1	0.6227 (3)	0.5359 (3)	0.3281 (2)	0.0338 (5)	
C2	0.8010 (3)	0.6555 (3)	0.4224 (2)	0.0342 (5)	
C3	0.8216 (3)	0.7875 (3)	0.5161 (2)	0.0371 (6)	
H3	0.9405	0.8616	0.5755	0.044*	
C4	0.6675 (3)	0.8101 (3)	0.5224 (2)	0.0356 (6)	
C5	0.4892 (3)	0.7045 (3)	0.4328 (2)	0.0352 (6)	
H5	0.3854	0.7222	0.4356	0.042*	
C6	0.4730 (3)	0.5752 (3)	0.3417 (2)	0.0320 (5)	
C7	0.7112 (4)	0.0619 (4)	0.1648 (3)	0.0537 (7)	
H7	0.6806	0.0272	0.2355	0.064*	
C8	0.7330 (4)	−0.0490 (4)	0.0772 (2)	0.0441 (6)	
C9	0.7774 (4)	0.0108 (4)	−0.0253 (2)	0.0460 (7)	
H9	0.7911	−0.0610	−0.0875	0.055*	
C10	0.8018 (4)	0.1739 (4)	−0.0378 (3)	0.0516 (7)	
H10	0.8335	0.2132	−0.1071	0.062*	
C11	0.7792 (4)	0.2771 (4)	0.0525 (3)	0.0530 (7)	
H11	0.7960	0.3885	0.0460	0.064*	
C12	0.7062 (5)	−0.2262 (4)	0.0915 (3)	0.0683 (9)	
H12A	0.7274	−0.2835	0.0214	0.102*	
H12B	0.7972	−0.2084	0.1665	0.102*	
H12C	0.5769	−0.3007	0.0957	0.102*	
N1	0.9729 (3)	0.6445 (3)	0.4219 (2)	0.0495 (6)	
N2	0.6903 (3)	0.9444 (3)	0.6245 (2)	0.0461 (6)	
N3	0.2906 (3)	0.4686 (3)	0.24334 (19)	0.0398 (5)	
N4	0.7332 (3)	0.2180 (4)	0.1493 (2)	0.0549 (7)	
O1	0.9718 (3)	0.5443 (3)	0.3360 (3)	0.0885 (9)	
O2	1.1190 (3)	0.7419 (4)	0.5074 (2)	0.0792 (8)	
O3	0.8528 (3)	1.0446 (3)	0.69676 (19)	0.0630 (6)	
O4	0.5489 (3)	0.9540 (3)	0.6359 (2)	0.0670 (6)	
O5	0.2505 (3)	0.5433 (3)	0.1711 (2)	0.0799 (7)	
O6	0.1909 (3)	0.3122 (3)	0.2365 (2)	0.0673 (6)	
O7	0.5902 (3)	0.4101 (3)	0.24116 (17)	0.0518 (5)	
H4A	0.712 (6)	0.291 (5)	0.205 (4)	0.093 (12)*	

### Atomic displacement parameters (Å^2^)

**Table:**

	*U*^11^	*U*^22^	*U*^33^	*U*^12^	*U*^13^	*U*^23^
C1	0.0374 (13)	0.0393 (13)	0.0313 (12)	0.0242 (11)	0.0115 (10)	0.0080 (10)
C2	0.0287 (12)	0.0393 (13)	0.0389 (13)	0.0200 (10)	0.0124 (10)	0.0079 (10)
C3	0.0301 (13)	0.0386 (13)	0.0358 (13)	0.0136 (10)	0.0085 (10)	0.0050 (10)
C4	0.0384 (14)	0.0339 (12)	0.0345 (12)	0.0186 (11)	0.0138 (11)	0.0031 (10)
C5	0.0361 (13)	0.0396 (13)	0.0381 (13)	0.0246 (11)	0.0141 (11)	0.0084 (11)
C6	0.0310 (12)	0.0372 (12)	0.0302 (12)	0.0203 (10)	0.0074 (10)	0.0063 (10)
C7	0.0408 (16)	0.072 (2)	0.0438 (15)	0.0268 (14)	0.0152 (12)	0.0057 (14)
C8	0.0328 (13)	0.0488 (15)	0.0427 (15)	0.0188 (12)	0.0067 (11)	0.0002 (12)
C9	0.0453 (15)	0.0493 (15)	0.0403 (14)	0.0272 (13)	0.0078 (12)	−0.0050 (12)
C10	0.0584 (18)	0.0547 (17)	0.0434 (15)	0.0330 (14)	0.0112 (13)	0.0048 (13)
C11	0.0496 (17)	0.0504 (16)	0.0557 (18)	0.0304 (14)	0.0038 (14)	−0.0005 (14)
C12	0.067 (2)	0.062 (2)	0.076 (2)	0.0307 (17)	0.0222 (18)	0.0207 (17)
N1	0.0350 (13)	0.0548 (14)	0.0596 (15)	0.0254 (11)	0.0136 (11)	0.0064 (12)
N2	0.0503 (14)	0.0424 (12)	0.0428 (12)	0.0233 (11)	0.0148 (11)	0.0009 (10)
N3	0.0386 (12)	0.0480 (13)	0.0360 (11)	0.0269 (11)	0.0076 (9)	0.0048 (10)
N4	0.0416 (13)	0.0639 (16)	0.0530 (15)	0.0316 (12)	0.0076 (11)	−0.0137 (13)
O1	0.0459 (13)	0.0855 (16)	0.114 (2)	0.0365 (12)	0.0156 (13)	−0.0352 (15)
O2	0.0415 (12)	0.116 (2)	0.0693 (15)	0.0458 (13)	−0.0011 (11)	−0.0092 (14)
O3	0.0572 (13)	0.0555 (12)	0.0524 (12)	0.0195 (10)	0.0083 (10)	−0.0134 (10)
O4	0.0646 (14)	0.0736 (14)	0.0663 (14)	0.0430 (12)	0.0233 (11)	−0.0078 (11)
O5	0.0700 (16)	0.0835 (16)	0.0718 (15)	0.0343 (13)	−0.0089 (12)	0.0310 (13)
O6	0.0546 (13)	0.0478 (13)	0.0684 (14)	0.0122 (10)	−0.0058 (10)	0.0049 (10)
O7	0.0504 (11)	0.0585 (12)	0.0472 (11)	0.0367 (10)	0.0068 (9)	−0.0093 (9)

### Geometric parameters (Å, º)

**Table:**

C1—O7	1.251 (3)	C9—C10	1.371 (4)
C1—C2	1.432 (3)	C9—H9	0.9300
C1—C6	1.434 (3)	C10—C11	1.357 (4)
C2—C3	1.372 (3)	C10—H10	0.9300
C2—N1	1.444 (3)	C11—N4	1.318 (4)
C3—C4	1.366 (3)	C11—H11	0.9300
C3—H3	0.9300	C12—H12A	0.9600
C4—C5	1.394 (3)	C12—H12B	0.9600
C4—N2	1.439 (3)	C12—H12C	0.9600
C5—C6	1.351 (3)	N1—O1	1.205 (3)
C5—H5	0.9300	N1—O2	1.218 (3)
C6—N3	1.461 (3)	N2—O4	1.217 (3)
C7—N4	1.326 (4)	N2—O3	1.230 (3)
C7—C8	1.375 (4)	N3—O6	1.199 (3)
C7—H7	0.9300	N3—O5	1.205 (3)
C8—C9	1.377 (4)	N4—H4A	0.93 (4)
C8—C12	1.491 (4)		
			
O7—C1—C2	127.4 (2)	C8—C9—H9	119.3
O7—C1—C6	120.7 (2)	C11—C10—C9	119.0 (3)
C2—C1—C6	111.90 (19)	C11—C10—H10	120.5
C3—C2—C1	123.2 (2)	C9—C10—H10	120.5
C3—C2—N1	116.0 (2)	N4—C11—C10	119.4 (3)
C1—C2—N1	120.8 (2)	N4—C11—H11	120.3
C4—C3—C2	120.0 (2)	C10—C11—H11	120.3
C4—C3—H3	120.0	C8—C12—H12A	109.5
C2—C3—H3	120.0	C8—C12—H12B	109.5
C3—C4—C5	121.2 (2)	H12A—C12—H12B	109.5
C3—C4—N2	119.0 (2)	C8—C12—H12C	109.5
C5—C4—N2	119.7 (2)	H12A—C12—H12C	109.5
C6—C5—C4	117.4 (2)	H12B—C12—H12C	109.5
C6—C5—H5	121.3	O1—N1—O2	121.1 (2)
C4—C5—H5	121.3	O1—N1—C2	120.0 (2)
C5—C6—C1	126.1 (2)	O2—N1—C2	118.8 (2)
C5—C6—N3	118.5 (2)	O4—N2—O3	122.8 (2)
C1—C6—N3	115.36 (19)	O4—N2—C4	119.0 (2)
N4—C7—C8	120.7 (3)	O3—N2—C4	118.2 (2)
N4—C7—H7	119.6	O6—N3—O5	123.3 (2)
C8—C7—H7	119.6	O6—N3—C6	119.3 (2)
C7—C8—C9	116.5 (3)	O5—N3—C6	117.4 (2)
C7—C8—C12	121.8 (3)	C11—N4—C7	123.0 (2)
C9—C8—C12	121.8 (2)	C11—N4—H4A	115 (2)
C10—C9—C8	121.5 (2)	C7—N4—H4A	122 (2)
C10—C9—H9	119.3		
			
O7—C1—C2—C3	−178.4 (2)	C7—C8—C9—C10	1.2 (4)
C6—C1—C2—C3	1.9 (3)	C12—C8—C9—C10	−180.0 (3)
O7—C1—C2—N1	2.7 (4)	C8—C9—C10—C11	−0.8 (4)
C6—C1—C2—N1	−177.0 (2)	C9—C10—C11—N4	−0.4 (4)
C1—C2—C3—C4	−0.2 (4)	C3—C2—N1—O1	−172.7 (3)
N1—C2—C3—C4	178.7 (2)	C1—C2—N1—O1	6.3 (4)
C2—C3—C4—C5	−1.9 (4)	C3—C2—N1—O2	5.0 (4)
C2—C3—C4—N2	177.4 (2)	C1—C2—N1—O2	−176.0 (3)
C3—C4—C5—C6	2.0 (4)	C3—C4—N2—O4	−173.7 (2)
N2—C4—C5—C6	−177.3 (2)	C5—C4—N2—O4	5.6 (4)
C4—C5—C6—C1	0.0 (4)	C3—C4—N2—O3	6.1 (4)
C4—C5—C6—N3	−177.0 (2)	C5—C4—N2—O3	−174.6 (2)
O7—C1—C6—C5	178.5 (2)	C5—C6—N3—O6	−112.1 (3)
C2—C1—C6—C5	−1.8 (3)	C1—C6—N3—O6	70.5 (3)
O7—C1—C6—N3	−4.4 (3)	C5—C6—N3—O5	69.4 (3)
C2—C1—C6—N3	175.3 (2)	C1—C6—N3—O5	−108.0 (3)
N4—C7—C8—C9	−0.3 (4)	C10—C11—N4—C7	1.4 (4)
N4—C7—C8—C12	−179.1 (3)	C8—C7—N4—C11	−1.0 (4)

### Hydrogen-bond geometry (Å, º)

**Table:**

*D*—H···*A*	*D*—H	H···*A*	*D*···*A*	*D*—H···*A*
N4—H4*A*···O1	0.93 (4)	2.27 (4)	2.827 (4)	118 (4)
N4—H4*A*···O7	0.93 (4)	1.79 (5)	2.638 (4)	152 (4)
C5—H5···O2^i^	0.93	2.51	3.406 (4)	162
C10—H10···O3^ii^	0.93	2.55	3.220 (4)	129
C12—H12*B*···O3^iii^	0.96	2.56	3.414 (5)	149

Symmetry codes: (i) *x*−1, *y*, *z*; (ii) *x*, *y*−1, *z*−1; (iii) −*x*+2, −*y*+1, −*z*+1.
